# Prediction and Optimization of Process Parameters for Composite Thermoforming Using a Machine Learning Approach

**DOI:** 10.3390/polym14142838

**Published:** 2022-07-12

**Authors:** Long Bin Tan, Nguyen Dang Phuc Nhat

**Affiliations:** Institute of High Performance Computing (IHPC), A*STAR, 1 Fusionopolis Way, #16-16, Connexis North Tower, Singapore 138632, Singapore; dpnhat.nguyen@u.nus.edu

**Keywords:** carbon fiber, woven composites, thermoforming, machine learning, optimization, artificial neural network, thermoplastics

## Abstract

Thermoforming is a process where the laminated sheet is pre-heated to the desired forming temperature before being pressed and cooled between the molds to give the final formed part. Defects such as wrinkles, matrix-smear or ply-splitting could occur if the process is not optimized. Traditionally, for thermoforming of fiber-reinforced composites, engineers would either have to perform numerous physical trial and error experiments or to run a large number of high-fidelity simulations in order to determine satisfactory combinations of process parameters that would yield a defect-free part. Such methods are expensive in terms of equipment and raw material usage, mold fabrication cost and man-hours. In the last decade, there has been an ongoing trend of applying machine learning methods to engineering problems, but none for woven composite thermoforming. In this paper, two applications of artificial neural networks (ANN) are presented. The first is the use of ANN to analyze full-field contour results from simulation so as to predict the process parameters resulting in the quality of the formed product. Results show that the developed ANN can predict some input parameters reasonably well from just inspecting the images of the thermoformed laminate. The second application is to optimize the process parameters that would result in a quality part through the objectives of minimizing the maximum slip-path length and maximizing the regions of the laminate with a predesignated shear angle range. Our results show that the ANN can provide reasonable optimization of the process parameters to yield improved product quality. Overall, the results from the ANNs are encouraging when compared against experimental data. The image analysis method proposed here for machine learning is novel for composite manufacturing as it can potentially be combined with machine vision in the actual manufacturing operation to provide active feedback to ensure quality products.

## 1. Introduction

In the last decade, machine learning has been utilized across different industries. In engineering and manufacturing, a properly trained artificial neural network (ANN) can help to develop design guidelines or provide optimized solutions that would substantially reduce the design cycle time. In the first application, the slip-path length contour images from hundreds of finite element thermoforming analyses are used as inputs and the ANNs are trained to predict the original process parameters based on new images from unseen cases. Knowing approximately the actual process parameters that formed the part can provide a causal link insight to help develop design rules and allow an experienced engineer to propose improvements on the manufacturing processes so as to mitigate part defects. In the second application, another ANN is used for process parameter optimization in order to minimize the maximum slip-path length (indicator for surface defect) and to maximize the shear angle coverage (indicator for formability) for ply angles between 40 to 50 degrees of the formed laminate part. The second criterion is in tandem with promoting the draping of the laminate by ply shear mechanism rather than out-of-plane bending which will cause wrinkling.

## 2. Background

In this work, the finite element analysis software AniForm^TM^ [1] is used to simulate thermoforming of woven carbon fiber thermoplastic laminate under various process conditions. Each simulation can take between 1 to 2 h which is a time-consuming process. The motivation is to apply artificial neural network (ANN) to expeditiously predict better process parameters so as to reduce design cycle time. This process can help engineers explore the design space much faster, help develop correlations and design guidelines for thermoforming and in deriving an optimal set of process parameters that would create a better-quality part.

### 2.1. Artificial Neural Network and Convolutional Neural Network

Neural network in computer science is an attempt to approximate the biological neural network of human brains. Its application in the field of machine learning is vast and can include natural language processing, object recognition, data analytics and many others.

An ANN is a system of many computing devices called neurons. Usually, an ANN consists of multiple neuron layers: an input layer followed by hidden layers and a final output layer. In a feed-forward NN (FFNN), the neurons in a layer only receive information from neurons in previous layers and send information to neurons in subsequent layers. Typically, only the neurons in two consecutive layers are connected. This connection is weighed and comes with a weight value. Despite the great variety of layer types, most neurons would perform the multiply and accumulate (MAC) operation on the data received from other neurons and then apply a non-linear function on the result. In this paper, two types of FFNN are developed. The first is the fully connected neural network (FCNN), whereby each neuron in layer *i* is connected to all neurons in layer *i* + 1, and the defining parameters for each FC layer are the number of output neurons. The second is the convolutional neural network (CNN) that is commonly used in the field of image classification, whereby ANNs with many convolutional blocks (Conv) extract features, followed by FC layers that would perform prediction using the extracted feature vector.

In a Conv layer, a sliding filter is applied to perform MAC operations on a region of the input and stores the result in the output tensor, called a feature map. The filter will take a stride, which can be multi-dimensional, to the next region after each MAC operation. Finally, an activation function is applied at each position of the feature map to compute the output of the Conv layer. As sliding filters can stride over multiple dimensions, they can be used to extract inter-dimensional and/or spatial information from the input. The defining parameters of a Conv layer include the filter size and the stride.

**Figure 1:**
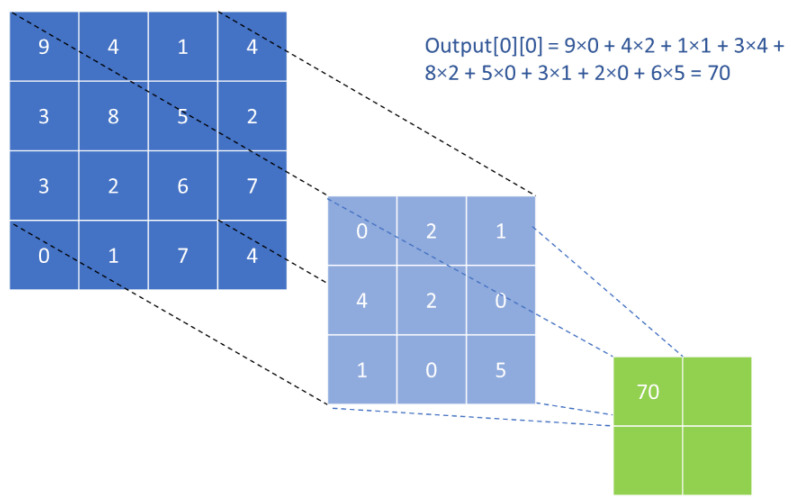
Example of convolution operation.

Figure 1 shows an example of how a sliding filter works. The input source is a 2D array while the filter is a 3 × 3 array. Each element in the filter represents a weight. When the filter is applied on a region of the input source (overlapping region), its product with the corresponding element in the filter is accumulated. Essentially, the filter is a weight matrix used to carry out MAC operations on a region of the input tensor.

### 2.2. ANN Applications in Manufacturing Research

To the best of the authors’ knowledge, almost all applications of ML in manufacturing thus far belong to the category of supervised learning whereby the training dataset is fully labelled. How the data are generated is very specific to the problem investigated, and for each engineering field, there are different considerations for data. As early as 2003, applications of ML in manufacturing were present. In [2], an overview of basic ANN applications in polymer composites was conducted. Methodologies reviewed in this paper were used to predict fatigue life, wear performance and dynamic mechanical properties for the material and its response under combined loading situations. Fully connected (FC) networks, using backpropagation training algorithms, were primarily used for these earlier works targeting direct problems.

For more recent and novel approaches, Chang et al. [3] developed an ANN with the desired product dimensions as modeling inputs to predict parameters for the thermoforming of polymeric foam sheets, which is an inverse problem. This work employed the use of a small FC network with layer sizes $\left(6,x_{0},x_{1},6\right)$ and tanh activation functions. The inputs to the ANN are the thickness measured at six locations while the outputs are the processing parameters: heater temperature, plug displacement, vacuum time, vacuum pressure, plug velocity and plug material type. Multiple experiments were conducted and the optimal network size was found to be (6, 5, 2, 6). Prediction for six parameters is a rare sight, especially when the reported dataset is small (just 40 cases). The data generation method is based on *single*-*parameter variation*, such that only one variable changes while others remain constant. This greatly simplifies the problem as the data are not coupled and therefore do not effectively allow the ANN to learn the relationships between parameters. As the root-mean-square errors (RMSE) or loss values for all processing parameters were under 0.01, an acceptable accuracy was reported and the method is deemed suitable for the specific problem.

Simoncini et al. [4] used ANN to predict the maximum stress of tensioned ABS coupons undergoing the IR-heating process similar in thermoforming. A FC network with three layer sizes (5, 11, 1) was used whereby the five inputs are: operating heat source, crosshead speed, thickness of sample, distance from heat source and elongation of sample. The sole output was the stress–strain response of the coupon. The dataset only comprises 24 cases so it is inconclusive as to how good the ANN was at generalization.

Leite et al. [5] studied the application of ANN for the vacuum thermoforming process. Simulation data were generated and a FCNN was used. The five inputs comprise process parameters: heating time, electric heating power, mold actuator power, vacuum time and vacuum pressure. The outputs are dimensional deviation in height, deviation of the diagonal length, geometric deviation of flatness and of the side angles. First, the ANN was trained to predict the abovementioned deviation groups so as to obtain a set of values representative of the final product geometry. The authors then proceeded to optimize a target function that combines the four metric functions, with the ANN serving as a proxy that helped with mapping parameters to the target function, that can be understood as a criterion to meet for the part design.

Zobeiry et al. [6] studied the application of theory-guided ML (TGML) on laminate damage characterization, which is a direct problem. In total, 10,000 simulations were conducted. The ANN took in five input parameters: Young’s modulus, initial damage slope angle and the strains at three different points of the stress–strain curve with strain-softening response based on MAT81 material model in LS-DYNA. The training process was carried out with a validation split of 70/30 from the dataset and the mean squared error (MSE) was the loss function used for the evaluation of training quality. Four fully connected NNs were trained to each consecutively to predict four parameters: overall fracture energy, peak stress, slope of the damage function and strain-softening parameter. Knowledge in mechanics was evoked to build the chain of NNs where the output of one NN is used to guide the prediction of the next parameter. An advantage of this method is that the predicted data are uncoupled. Therefore, each NN can learn more effectively. More specifically, each NN can have a dedicated input vector and their weights can be trained to model one theoretical function only. However, the number of required simulations is huge, likely making the method infeasible to other engineering problems which may have limited data.

Nardi et al. [7] developed an ANN to predict final part attributes using the input temperatures of the thermoforming process, comparing the results to those from analytical and finite-element modeling (FEM) methods. The FC network has the architecture (3, 5, 3) with three layers and three output neurons. The small architecture was sensible as having more neurons can lead to overfitting or long training time. The Bayesian-regularization method, different from many other ANN approaches that used some variants of backpropagation, was used. It was not explained why the Sigmoid activation function was chosen, instead of the more popular ReLU function that can help gain sparsity when computing and better prevent vanishing gradients.

More recently, Humfeld et al. [8] studied the problem of optimizing air temperature cycle in the autoclave for composite processing. Due to uncertainties including tool placement, convective boundary conditions vary in each run. As a result, temperature histories in some of the parts may not conform to process specifications due to under-curing or over-heating. Recurrent NNs, a class of NNs where data from past inputs are remembered, were used in a FC network. This memory of past data is taken into account when operating on the current input. Compared to FFNN, there is a feedback link in the RNNs. Data do not simply flow from the first to last layers but can be sent backwards when computing the next input. It has broad application in natural language processing due to its ability to analyze sequential data. In total, 100,000 simulations with eight input parameters comprising heat transfer BCs, thickness of composite part, thickness of tool and air temperature profiles were conducted. The validation split was 70/30. The ANN is used for inverse modeling to predict the bottom tool temperature and the center part temperature. Multi-objective optimization, using the same ANN, that predicts the probabilities of the temperatures on the composite being acceptable or not, is then performed. For each ANN simulation case, if the maximum part temperature and part temperature rate fall into a desirable range, *T_max_ < 185 °C*, *1 °C*/*min < T_part_ < 3 °C*/*min*, the case is classified as “pass”. The outputs to this FCNN would be the failure and pass probabilities whereby cases/conditions with high pass probability are chosen and a sorting step is used to find the most optimal one.

Wanigasekara et al. [9] extended their previous work, which built a direct model to predict output characteristics of thermoplastic composite laminates used in automated fiber placement machines and resolving it using inverse modeling for the same manufacturing problem. In the direct method, an ANN was used to predict four characteristics of thermoplastic composite laminates, namely, elastic modulus, short-beam strength/inter-laminar shear strength, maximum flexural stress and maximum flexural strain, based on four inputs, i.e., the deposition rate, consolidation force, hot gas torch temperature and its corresponding nip-point temperature. The inverse problem studied here simply reverses the flow of prediction. The authors showed that from a small set of 28 experimental data, virtual data can be generated [10] whereby the training set for the inverse model consisted of predictions made by the direct model, excluding outliers, in addition to the original data. This approach showed a relationship between the direct and inverse models where one can be used to train the other. It was inferred that as more data are available, both models can be improved with little modification to the training pipeline.

Melaibari et al. [11] applied neural networks to predict the GO-CuO/water-EG hybrid nanofluid viscosity that is found from the process of loading graphene oxide and copper oxide nanoparticles into ethylene glycol-water. Two methods were studied and compared: neural networks and response surface methodology (RSM). The ANNs comprised three layers, with the architecture (3, 10, 1). The three inputs are temperature, mass fraction and shear rate and the output value is the viscosity. Additionally, the Sigmoid activation function was used. However, the training setup such as the dataset size, optimizer, etc., was not reported. On the other hand, the RSM uses the same three inputs to form a polynomial predicting the viscosity. The polynomial consists of 19 parameters that are learned. From the experiments, the ANN achieved a mean square error of 0.0125 while RSM reached 0.166. This is quite sensible, considering that the ANN had 3 × 10+10 × 1=40 parameters to fine-tune, almost double that of the RSM polynomial.

In many of these works, the neural networks used were small FC networks and the main interest came from the workflow which can be used as a guideline for other manufacturing ML problems.

In the domain of quality control, a few papers have introduced the use of CNN. Nuria et al. [12] incorporated CNN into a computer vision quality control system for the sealing of thermoforming food packages. In particular, the system contains multiple components, from a vision software that processes images from the camera to a CNN that makes the prediction of accepting or rejecting the package. Multiple neural network architectures were used: five ResNet configurations, three VGG configurations and two DenseNet configurations. Each architecture would be trained on all five datasets separately. This is to find which dataset fits the real-word scenario the most. In total, there are 2978 training images and 628 validation images. For each network, it can be trained from scratch or trained from a set of weights pre-trained on ImageNet—an extensive dataset for object classification, first described in [13]. The input to the CNN is a mono-infrared (read black and white) image, while the output is a binary decision of accept/reject. For this type of prediction, the output could either be one or two neurons. It is unclear how pre-trained models are trained or used for prediction, since ImageNet contains RGB images that have different dimensions from the mono ones. The results shown were very promising. All models achieved an accuracy of at least 93%. Pre-trained DenseNet161 proved to be the best with 99%. This work proved the usefulness of CNNs in the real-world setting. It has also demonstrated that using pre-trained models can be helpful. This can serve as a guideline for other applications of CNN in manufacturing to create a system of both computer vision software and convolutional neural networks.

## 3. Model Overview and Material Properties

**Figure 2:**
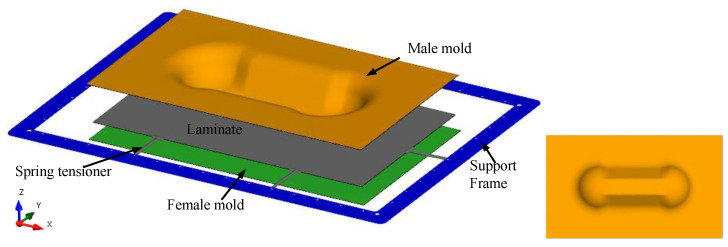
Thermoforming setup (**left**); Double-dome mold geometry—bottom view (**right**).

Figure 2 shows the model setup for the thermoforming process analysis. The molds are of a double-dome geometry. The support frame and mold tools are rigid and the underlying fabric-reinforced thermoplastic laminate comprises two layers of plain-woven carbon fiber prepregs that are each 0.3 mm thick. The laminate is gripped using spring tensioners and suspended within the supporting frame to transport it from the heating stage to the molding stage where the slightly tensioned laminate will be placed in between the upper and lower molds before being pressed to form the part. Tensioners are typically used to reduce the amount of laminate sagging and improve laminate alignment with the mold when the thermoplastic composite has been heated.

The thermoforming behavior of the laminate is mathematically captured using various material models. Essentially, the mechanisms observed in composite laminate forming are intra-ply in-plane shear and bending and tool–ply and ply–ply interfacial slippage. Table 1 shows the ply properties used for the thermoforming analysis in AniForm^TM^.

The isotropic elastic and the cross-viscosity models are jointly used to predict the in-plane and bending behaviors of the plies. The cross model is a shear-rate-dependent viscosity model as found in the work of Macosko [14]. It is used to model power law type of response with a viscosity plateau region at low and high shear rates, respectively. The mathematical equations below describe the cross model:

$$\eta \left(\dot{\gamma }\right)=\frac{\eta_{0}-\eta_{\infty }}{1+m{\dot{\gamma }}^{1-n}}+\eta_{\infty }$$

and

$$\sigma =\frac{2\eta (\dot{\gamma })}{J}D$$

where **σ** is the Cauchy stress, **D** is the rate of deformation tensor and *J* is the Jacobian of the deformation gradient.

The Mooney–Rivlin model is a hyperelastic model, suited to model the response of rubber-like materials. The general expression for strain energy **W** is given as:

$$W=\overset{n}{\underset{i=0,j=0}{\sum }}C_{ij}\left(I_{1}-3\right)(I_{2}-3)$$

with *C_ij_* material constants and *I*_1_ and *I*_2_ the strain invariants. AniForm^TM^ uses the two parameter Mooney–Rivlin model, where *C*_01_ and *C*_10_ can be set for *n* = 1, *C*_00_ = *C*_11_ = 0. The Cauchy stress, **σ**, is hence:

$$\sigma =\frac{1}{J}.\left(2C_{10}\left(B-I\right)-2C_{01}\left(B^{-1}-I\right)\right)$$

where ***B*** is the Cauchy–Green deformation tensor and *J* is the Jacobian of the deformation gradient.

A mixed model that combines the models in parallel is used to describe the overall deformation mechanism. The total stress response is equal to $\sigma =\sum_{i=1}^{n}\upsilon_{i}\sigma i$, where each basic model stress tensor, **σ*_i_***, can be scaled by a weight. In our modeling, we assume equal weightage between the elastic and viscous response, i.e., υ_1_ = υ_1_ = 1.

### 3.1. Part Profile

A geometry for the part profile has to be selected for this machine learning work. The double-dome geometry, with doubly curved regions of steep walls and small radii, is chosen as it is widely used by research groups [15,16,17] as a suitable benchmark for the investigation of forming behavior. The groups aim to support the development of reliable and robust simulations for forming processes. The benchmark metrics for comparison include shear angles, draw-in and possible presence of wrinkles. The chosen testing material comprised balanced plain weave (BPW), balanced twill weave (BTW) and UBTW Twintex comingled glass/PP fabric laminate.

For greater insights on the formability response of plain-woven fabric-reinforced thermoplastics, Rietman et al. [17] compared the results of AniForm^TM^ simulations of the double-dome (DD) geometry with published results. The authors pointed out that different laminate orientations alone would lead to completely different deformation results, which include the distribution of shear angle. It is with this consideration that our work also utilizes the double-dome geometry so as to provide insights on optimization and also gain wider readership and application.

### 3.2. Thermoforming Parameters Studied

Five process parameters were investigated. These include (1) laminate orientation, (2) spring stiffness of tensioners, (3) preload of grip tensioners, (4) forming/press Rate and (5) grip size. The effect of tool and laminate temperatures could not be effectively investigated as temperature-dependent ply properties were not available. The parameter values were varied to run a total of 200 Aniform^TM^ simulations. The values corresponded to practical thermoforming process parameter ranges and are shown in Table 2. It is noted that the run cases are not a full factorial space of the available parameters shown in Table 2.

**Figure 3:**
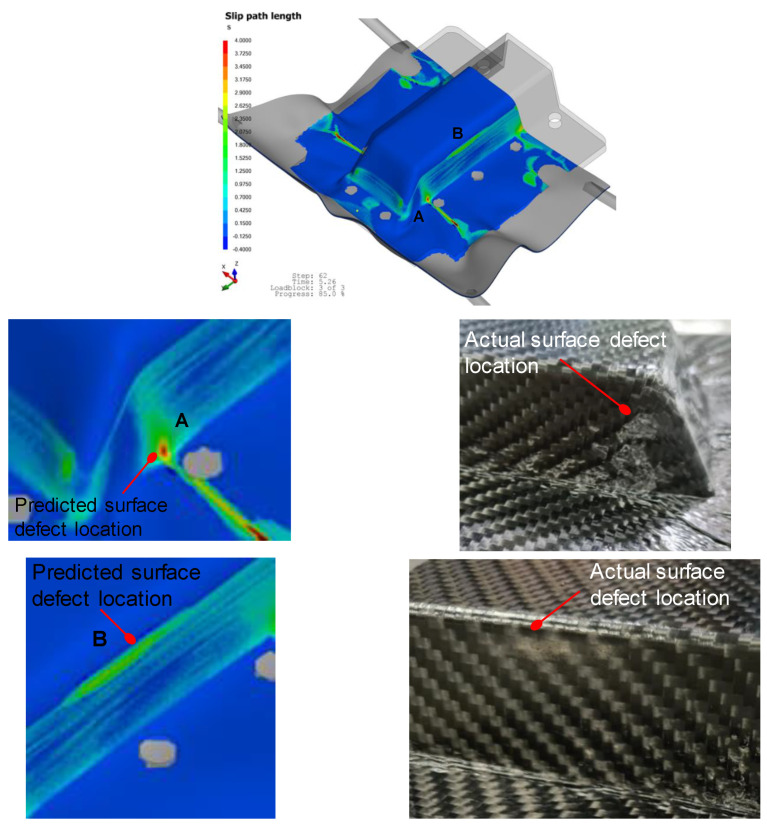
Good correlation of matrix smearing surface defect with slip-path length indicator.

**Figure 4:**
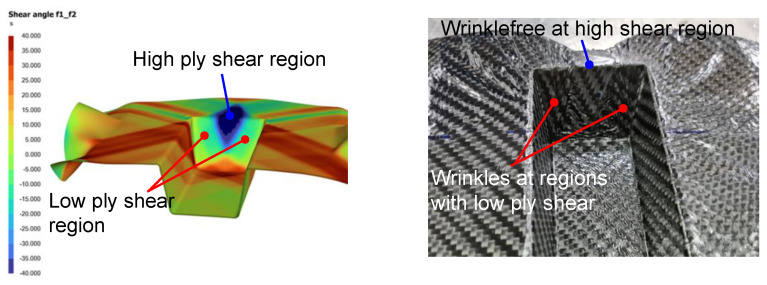

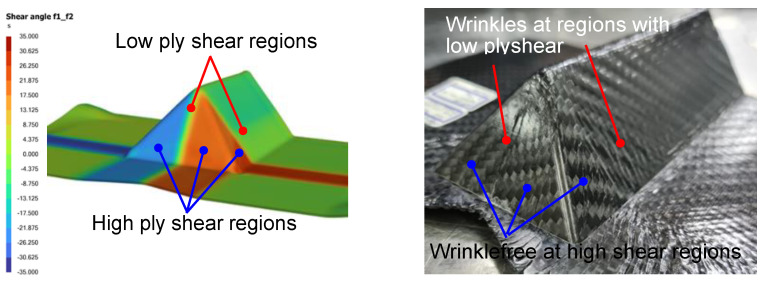
Good correlation of wrinkle formation with low ply shear angle.

Metrics such as the slip-path length and intra-ply shear angle results were used to evaluate the quality of the thermoformed part. The slip-path length is defined as the total slip that was encountered by a certain point at the tool–ply interface as the laminate needs to slide along the tooling during press forming [18]. The magnitude of this length could give an indication of the duration a particular region was exposed to a colder tooling surface, which will give a higher possibility of surface defect as shown by Figure 3, where in-house experiments and simulations, for the thermoforming of four ply 2 × 2 twill carbon fiber-reinforced thermoplastics, show positive correlation between the slip-path length and the location of matrix smearing or optical defect. This defect is caused by excessive traction and slippage of the solidifying laminate with the cooling surface. Figure 4 shows experimental verification that regions of the laminate with higher ply shear will less likely experience wrinkle formation. In the top pictures of Figure 4, the side regions of the thermoformed wall showed wrinkles upon forming, which correlates to lower ply shear angles at that region. Similarly, in the bottom pictures of Figure 4, the two sides of the triangular wall surface have higher ply shear resulting in a smoothly formed part while wrinkles were observed close to the centerline of the triangular surface which corresponds to a lower ply shear angle at that region.

**Figure 5:**
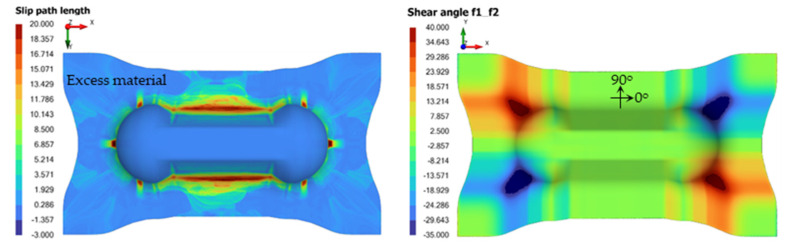
Example of slip-path length (**left**) and ply shear angle (**right**) distribution on the formed laminate.

Figure 5 (left) shows an example of the slip-path length and ply shear angle distribution on the double-dome part after thermoforming simulation. Higher slip-path lengths (as shown by the red regions) are typically near the transition from the vertical wall of the part to the excess material, with values as high as 15 to 20 mm. The location of high shear angles typically depends on the ply orientation relative to the mold. Figure 5 (right) shows the shear angle resulting from a 0 degree laminate. The maximum shear angles could reach +/−40 degrees.

## 4. ANN Training Methodology

### 4.1. Image Data Preprocessing

Machine learning is the process of learning a predictor to correctly predict a data space with the objective being either a regression (real number) task or a classification (discrete sets) task. Many learning paradigms have been proposed over the years. Non-ANN methods are specifically designed with restrictive assumptions in mind. They can excel with data spaces that fit their assumptions, but are difficult, if not impossible, to predict data with complicated distributions. Some important reasons why ANNs are used so widely today is that virtually no assumptions about the data space are made and that they are capable of capturing nuances from the data and provide nonlinear predictions.

**Figure 6:**
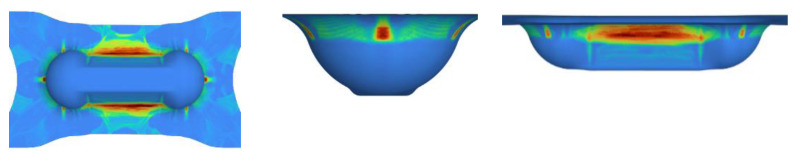
Bottom (**left**), front (**middle**) and side (**right**) views of SPL distribution of the double-dome part from AniForm^TM^ simulation.

The first part of this project explores the possibility of predicting process parameters based on the product images from finite element (FE) simulation contours similar to those in Figure 5. Although FE simulation contours were evaluated in our study, this does not exclude the use of images from actual formed physical products. In our study, the slip-path length (SPL) contour images are collected as input data for machine learning. To maximize the visual information gained, multiple views of the same case run are collected as shown in Figure 6, with the bottom view giving an overview of the SPL distribution and approximate location with high values, while the front and right views give more details of the SPL distribution on the sides of the laminate.

Before being processed by the ANNs, the images are normalized. The normalization step would convert pixel values into values between 0 and 1. As the original images have very high resolution, using these images would require extensive computational resources for ANN training. Therefore, they are rescaled to 336×336 pixels which allowed for good results while not requiring too much computational memory. In some cases, it is possible to rescale the images to 224×224 pixels and still achieve good results. Since the SPL distribution is represented by a color contour, it is appropriate to represent an input image as a 3D array with dimensions C×W×H, with *W* and *H* referring to the *width* and *height* of the pixel location while *C* refers to the number representing the R-G-B channel color. The area surrounding the laminate is just a white plain with each pixel having a RGB value of (0, 0, 0).

### 4.2. ANN Architecture in Inverse Modeling

**Figure 7:**
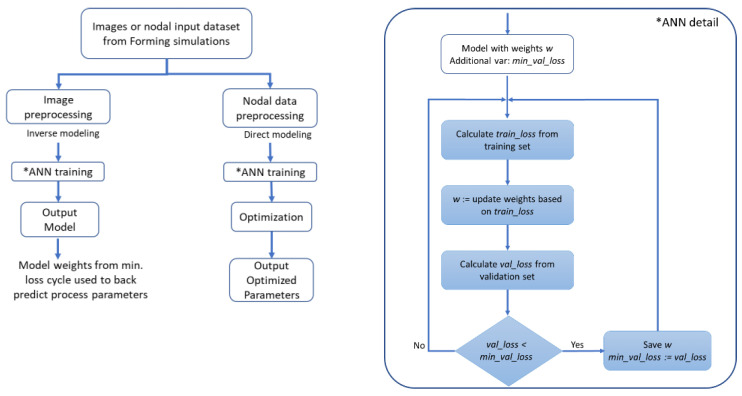
Data processing flow chart.

The data processing flow chart for our work is shown in Figure 7. The learning rate and batch size for all the ANNs are set to 0.001 and 8, respectively. These hyperparameters are selected based on the better convergence of training loss, after rounds of model testing with different settings.

The usage of CNNs is not limited to processing square images, but it is the most convenient in this study as one can anticipate the output of every convolutional layer to be a square image as well. One can also opt for a grayscale image if there is no color information. The workflow involved 2 stages: image processing to extract features and use of the features for regression. For the feature extraction phase, a strong CNN is used. Implementations of many popular CNNs can be found at Pytorch Vision GitHub repository [19]. These CNNs have been used widely in AI research and their performances have been well-documented. Originally, they were benchmarked based on the task of object classification and the results are summarized in [20]. Their applications spanned multiple fields, mostly in computer vision, such as in object detection [21] and facial recognition [22]. In the field of manufacturing, Nuria et al. [12] showed that CNNs can be used in quality control. Even though these models were developed for image classification, it is easy to modify them to perform regression. The only modifications are the input and output dimensions.

The AlexNet [23] and the ResNext-101 (variant of ResNet) CNNs are selected for the work. Essentially, AlexNet has a smaller threshold number of parameters/weights and is simpler in nature (normal convolutional block vs. residual block) compared to ResNext, so its learning power is limited. However, when a task is simple, the AlexNet model will require less time to train. The input to both networks is a 3D array C×W×H representation of an image. Table 3 shows the AlexNet architecture that was used in our study for the prediction of laminate orientation angle, and with the final layer having one output neuron.

**Figure 8:**
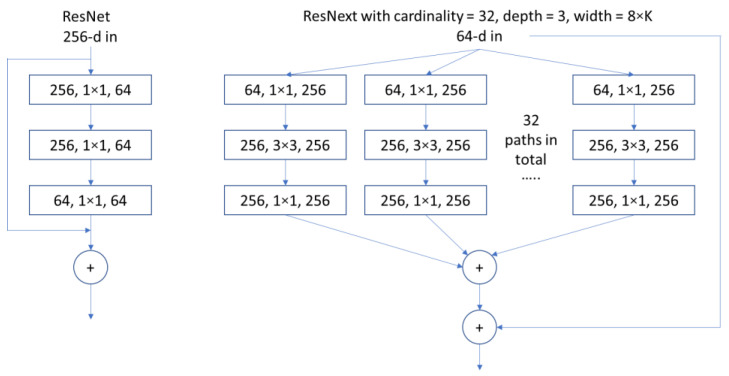
Residual block vs. ResNext block. Each layer is represented as the (number of input channels, filter size, and the number of output channels.

ResNet [24] stands for residual network where a residual block is being used instead of traditional Conv block similar to in AlexNet so that when the input is passed through the Conv layers, and at the final step, the original input can be added to the processed output to help the network gain back some information that could have been lost in the Conv process. ResNext [25] is a variant of ResNet that utilizes an enhanced kind of residual block. Figure 8 shows a normal ResNet block (with 3 weight layers) and the ResNext block architecture. The exact configuration used in this study is ResNext-101-32x8d where *cardinality* (the number of paths) is 32, *depth* (the number of operations in each path) is 3 for each block and the *width* (the number of output channels) of each block is a multiple of 8. The block illustrated in Figure 8 shows one of the many blocks used in the model.

To help with training, the dataset is normalized or rescaled to [0, 1]. The normalization conversion is given as follows:

- Laminate orientation, $x_{1}\to x_{1}{}^{1}=\left(x_{1}+45\right)/90$.
- Tensioner stiffness, $x_{2}\to x_{2}{}^{1}=x_{2}/2$.
- Preload, $x_{3}\to x_{3}{}^{1}=x_{3}/8$.
- Press rate, $x_{4}\to x_{4}{}^{1}=x_{4}/100$.
- Grip size, $x_{5}\to x_{5}{}^{1}=x_{5}/8$.

There are 2 approaches to building the predictor system. One is to use 1 ANN to predict all 5 output parameters, or to use 5 ANNs to each predict 1 parameter. The first approach is called MultiVar, while the second is called SingleVar. In MultiVar, a ResNext-101 model is used to predict all 5 process parameters. In SingleVar, an AlexNet model is used to predict the first parameter (laminate orientation angle) and 5 ResNext-101-32x8d models are used to each predict the remaining 5 parameters. Compared to other parameters, the laminate orientation angle is easier to predict using the bottom view and smaller image size, so using AlexNet in SingleVar would save a lot of training time due to the smaller number of parameters. Our preliminary experiments showed that Adam [26] was the preferred training algorithm other than stochastic gradient descent (SGD) [27]. The reason being with SGD, a longer training time would be required while yielding the same prediction accuracy, as also reported by Wilson et al. [28]. However, SGD has recently been shown to provide better generalization over adaptive optimizers [29]. From our point of view, the current work is a foundation step for future projects. Therefore, using Adam is a way to test the water.

To measure the accuracy of prediction, the concept of a *loss function* is needed. A loss function L measures the difference between the predictions by h and the labels of a set of data. In gradient descent—the baseline training algorithm for ANNs—the derivative of L is used to “guide” the learning parameters such that h heads towards a local minimum. In practice, the loss on a validation set $S^{\prime }$ (whose samples are not in the training set S) tells how well the training is going and how good the ANN is at predicting unseen data. Two types of loss function are used in this study:

- Mean absolute error (MAE):

$${\mathrm{MAE}}_{\left(h,S\right)}=\frac{1}{\left|S\right|}\underset{x_{i}\in S}{\sum }\left|h\left(x_{i}\right)-y_{i}\right|$$

- Mean squared error (MSE):

$${\mathrm{MSE}}_{\left(h,S\right)}=\frac{1}{\left|S\right|}\underset{x_{i}\in S}{\sum }{\left(h\left(x_{i}\right)-y_{i}\right)}^{2}$$

where h is a hypothesis, S is a set of $\left(x_{i},y_{i}\right)$ and $y_{i}$ is the label of $x_{i}$. When $y_{i}$ is a d-dimensional vector (representing multi-dimensional data), the difference $\left|h\left(x_{i}\right)\;-\;y_{i}\right|$ is the average of the differences at each of the d positions.

The MSE loss function was used during training, but the MAE was used during validation. This is because MAE is more comprehensible for engineering analysis purposes: for instance, an MAE loss of δ for a variable x would indicate that the prediction is in the range [x−δ,x+δ]. Using MSE loss would require complex conversion to get to the physical value, especially when the result is multi-dimensional. The advantage of using MSE loss during training is to help ensure the trained model has no outlier predictions with huge errors, since the MSE puts larger weight on these errors due to the squaring function.

### 4.3. Optimization of Slip-Path Length and Shear Angle in Direct Modeling

The first set of ANN models help us predict the maximum SPL and the proportions of in-range nodes. Subsequently, the optimization targets are to minimize the maximum SPL and to maximize the proportion of in-range nodes while minimizing the proportion of excessive nodes. The first problem is a standard minimization problem while the second one must be framed as the minimization of a particular function. Let the proportion of in-range and excessive nodes be $p_{1}$ and $p_{2},$ respectively. Then a target function can be:

$$F_{1}=-\left(p_{1}+\frac{\epsilon }{p_{2}+\epsilon }\right)$$

where ϵ is a small positive number. The function has a minimum value when $\left(p_{1},p_{2}\right)=\left[1,0\right]$. For comparison, the target function

$$F_{2}=-p_{1}$$

which ignores $p_{2}$ was also used.

For any target function, using Scipy library [30], we can call the built-in minimization/optimization function [31] to minimize the target value. The inputs to the target function, which have bounds of [0,1], would be the five process parameters as previously mentioned.

Since the optimization process requires an initial guess, this initial value was randomized. One could use the best case from the dataset, but it could lead the optimization to a wrong local minimum. And since a local minimum is returned, an easy way to find a good minimum is to run the process many times.

## 5. Results

This section summarizes the training statistics and validation accuracy of the ANNs described in the sections above. It is observed that not all process parameters are equally influential. This means that changing the values of some parameters would only cause very subtle differences to the AniForm^TM^ simulation results. The most influential parameter is the laminate orientation angle, as it determines where defects (high SPL values) could form. An example of a lower influential parameter is the grip size. This difference in parameter sensitivity does not affect the direct models, but greatly hampers the inverse models’ training to generalize. For all training graphs, the x-axis represents the number of training epochs passed and the y-axis represents the loss value. Each training graph would show the evolution of both the mean square error (MSE) and the mean absolute error (MAE) losses over the training cycles or epochs. For most cases, the losses will progressively reduce and converge to lower values or errors.

### 5.1. Inverse Modeling

**Figure 9:**
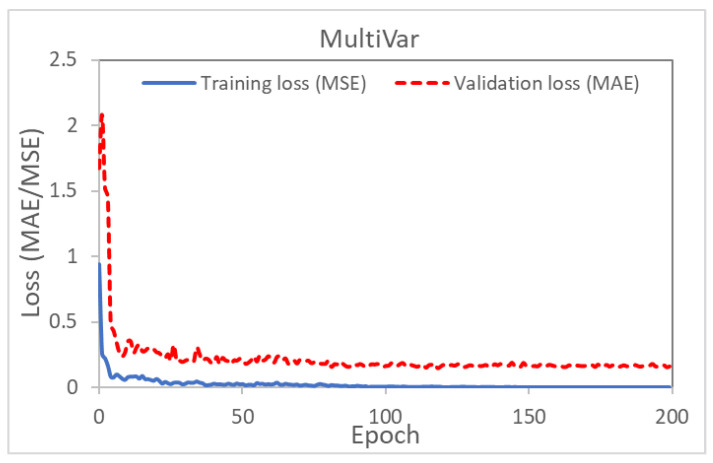
Graph of training and validation loss versus epoch (MultiVar prediction).

**Figure 10:**
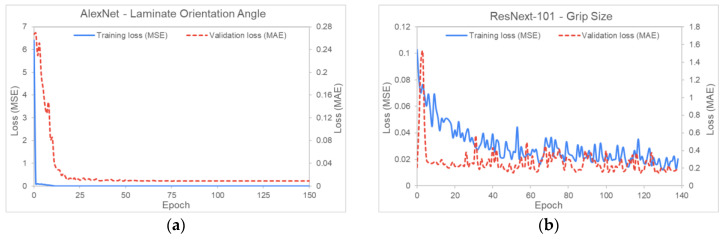
Training statistics for SingleVar for (**a**) laminate orientation and (**b**) grip size. Each graph denotes the network architecture and the parameter that it predicts.

Figure 9 and Figure 10 show the training MSE loss (blue line) and validation MAE loss (red line) for MultiVar and SingleVar regression. The losses in the MultiVar represents the average of all the five predicted outputs, while Figure 10a,b show the training graphs for laminate orientation and grip size. The training graphs for other process parameters are similar to those presented in the figures. The training and validation loss achieved for MultiVar regression is 0.001245 and 0.14693, respectively, while the losses for SingleVar (laminate orientation) are both lower than 0.0085. These values are comparable to those obtained by Chang et al. [3].

Table 4 summarizes the prediction accuracy of the ANNs on the validation set. The “Abs Error” columns refer to the average absolute difference (MAE) between the predicted outcomes and the actual labels. For practical manufacturing purposes, the range of error is acceptable because laminate rotation is usually performed by steps of 15 degrees, spring stiffness by 0.5 N/mm, preloads by 1N and grip sizes by 2 mm or more, on most exploratory test settings.

The laminate orientation angle has the most effect on part quality and it is also the easiest parameter to predict. The reason being the orientation angle would affect the overall shape of the thermoformed laminate, inclusive of the excess material. Ideally, the ANNs only need to extract information on the shape of the laminate’s edges in order to predict this parameter. In SingleVar, this ease of prediction can be seen clearly as the average error of prediction is less than 1 degree. For engineering purposes, this kind of error is effectively insignificant since laminate orientations employed for actual thermoforming are likely varied by angle steps of at least 15 degrees. The remaining parameters are less influential to the result images, so the validation loss is not as impressive, and these parameters also have a larger margin of error. The predicted parameters can be off by a considerable value, but the thermoformed laminate can still have the same SPL distribution because these parameters have a smaller effect than the predominant parameter which is laminate orientation. It is noted that MultiVar and SingleVar produced comparable results for these parameters.

Another observation is that the prediction accuracy for MultiVar, on the laminate orientation angle, is poorer than SingleVar. This can be attributed to the fact that the ANN has to extract features used for the prediction of all five parameters. Therefore, features related to this parameter might be affected or mixed with features that are used to predict other parameters. This adds another layer of complexity for MultiVar and thus can be an argument against the use of MultiVar in general. Furthermore, the MSE loss does not weigh individual differences at each position of the output vector. Consequently, during training, the orientation angle is not given more attention than other parameters, while this should have been the case. A solution for future work would be to have a weighted MSE loss function:

$$\;L_{2}\left(x,y\right)=\frac{1}{n}\sum \gamma_{i}{\left(x_{i}-y_{i}\right)}^{2}$$

where $\;x,y\in R^{n}$ and $\gamma_{i}$ are weight values corresponding to position i. This would ensure that more important parameters are emphasized and is an example of utilizing domain knowledge in the design of ANNs.

Furthermore, there are challenges that come with the image processing approach. Firstly, to the authors’ knowledge, there is no reported literature that argues that the chosen CNNs are the most appropriate for regression using multi-dimensional inputs. They were only tested for image classification problems. Secondly, this approach comes with a huge scalability problem. The input dimension of the images is very large—typically three RGB channels × three views × (336 × 336) pixels—leading to the CNN models having more than 80 million trainable parameters for the test set cases. As a result, training becomes both time consuming and computationally expensive, making it more difficult to fine-tune hyperparameters such as learning rate and batch size. Finally, image analysis can require an extensive setup to curate data and perform extensive testing, as in the case of Nuria et al. [12]. This is likely to present itself as a greater roadblock compared to fine-tuning.

**Figure 11:**
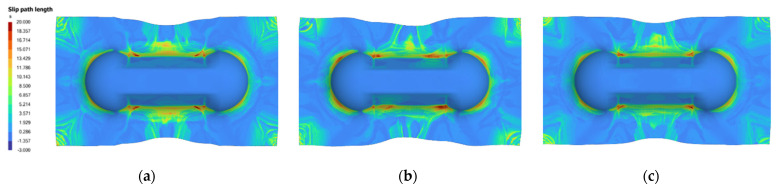
Example of good prediction of SPL (1): (**a**) Actual parameters: 45/45 laminate, S0.25 PL8, rate 16.67, point, grip 0; (**b**) MultiVar prediction: 50/50 laminate, S0.15 PL5, rate 45.48 (2.2 s), grip 4; (**c**) SingleVar prediction: 45/45 laminate, S0.27 PL6, rate 27.85 (3.6 s), grip 4.

**Figure 12:**
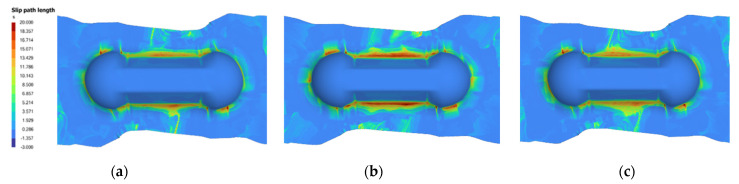
Example of good prediction of SPL (2): (**a**) Actual parameters: 15/15 laminate, S0.5 PL8, rate 33.33 (3 s), grip 8; (**b**) MultiVar prediction: 12/12 laminate, S1.18 PL5, rate 60.52 (1.7 s), grip 8; (**c**) SingleVar prediction: 15/15 laminate, S0.49 PL5, rate 26.63, grip 8.

**Figure 13:**
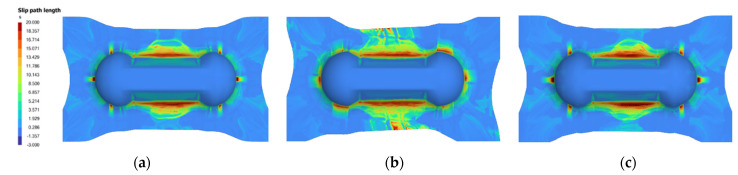
Example of moderately accurate prediction of SPL (3): (**a**) Actual parameters: 0/0 laminate, S1.0 PL2, rate 16.67 (6 s), grip 4; (**b**) MultiVar prediction: 8/8 laminate, S1.17 PL5, rate 51.81 (1.9 s), grip 6; (**c**) SingleVar prediction: 0/0 laminate, S1.04 PL5, rate 24.26 (4.1 s), grip 6.

In order to make a meaningful comparison, the predicted parameters are re-input into the AniForm^TM^ simulation to obtain the SPL contour plot to compare against the contour plot from the actual parameters used. Figure 11 and Figure 12 are examples of good predictions made by the ANNs. On the other hand, Figure 13 shows moderately accurate predictions where there is a greater deviation in the predicted parameters from the actual parameters: SingleVar prediction is not too far from the dataset image, while MultiVar prediction has more area with high SPL (dark red regions). The results show that the ANN is able to predict laminate orientation, spring stiffness, preload and grip size reasonably well. The MultiVar prediction accuracy is slightly poorer as typically much larger datasets would be required for the multi-variable training.

### 5.2. Optimizing Slip-Path Length

For the direct optimization problem, the ANNs only need to learn to predict an output (i.e., SPL) based on the fix process parameters; the problem becomes single-variate regression. Therefore, image processing is not necessary. This problem can be solved using a FCNN. The optimal architecture was found to be (5, 32, 16, 8, 1): input layer with 5 parameters, followed by 3 hidden layers with 32, 16 and 8 neurons, respectively, and the output layer with 1 neuron. The ANN simulation results from inverse modeling can be reused to form the dataset for this problem.

#### Prediction of Maximum Slip-Path Length (SPL)

**Figure 14:**
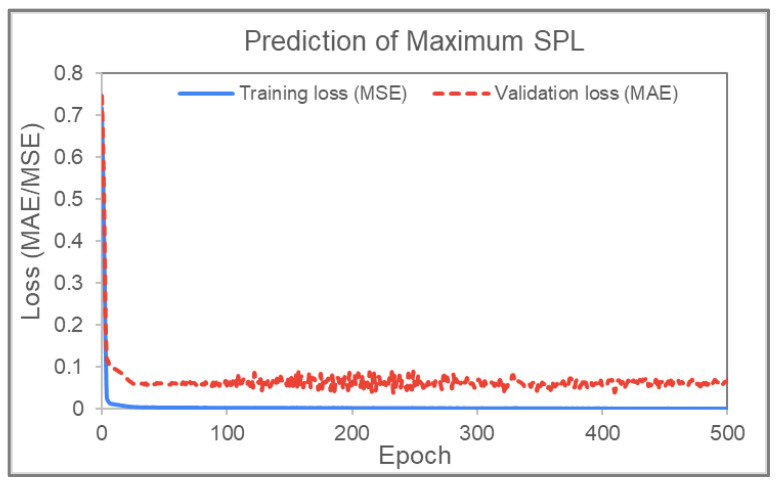
Graph of training and validation loss versus epoch (maximum SPL prediction).

The laminate response from the AniFrom^TM^ simulation is represented by a mesh of nodes, each with a corresponding SPL value. For the prediction of maximum slip-path length (SPL), the ANN uses a wrapper Python function to take in the five process parameters as inputs with normalized values of [0,1], and the output of the ANN would be the maximum SPL of all the nodes. Therefore, to train or validate the ANN, the maximum SPL found in the mesh of a case is used as a label for that case. It was found that the highest maximum SPL in the dataset is 26.448 but the maximum values in most of the cases are 23 or lower. The training graph for predicting maximum SPL is shown in Figure 14. The training and validation simulations were set to 1000 epochs (training cycles). The figure shows that the losses reduce significantly from 0.75 to around 0.1 within the first 5 epochs (or training cycles), and then from 0.1 to around 0.05 after 20 epochs. The error then fluctuates around this value for subsequent epochs. The validation loss seems higher than the training loss, but the two values should not be compared directly since the mean squared errors (with values below one) would undoubtedly yield smaller values. At the end of the neural network analysis, the parameter predictions from the cycle with the least error, and hence the most optimized case, are extracted. The best result achieved an MAE validation loss of 0.024877, while the lowest training error is 0.000911. This is comparable to the prediction accuracies achieved by Chang et al. [3].

Several optimization runs were initiated because it is likely that the solution would not be unique, considering the multiple parameters that are to be considered. The best predicted minimum and its corresponding process parameters are listed as:

*Max SPL: 14.409; Parameters: lam −34/−34, S0.18, PL1, rate 0.96 (103.87 s), grip 6.*

**Figure 15:**
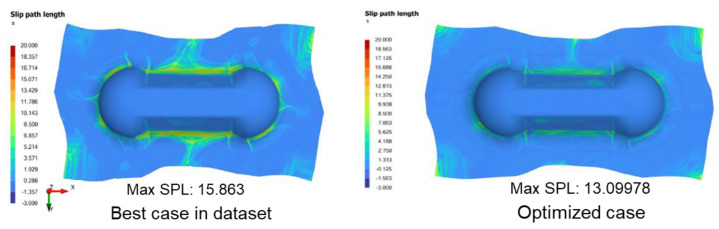
Comparison of SPL distribution for the best case in the dataset versus the optimized case with parameters derived from ANN.

For comparison, the lowest maximum SPL available in the dataset is 15.863. This corresponds to the process parameters: 30/30 laminate, S0.5 PL2, TT 150, rate 16.67 (6 s), grip 8. The optimized outputs from ANN are used to re-run the AniForm^TM^ simulation to quantitatively validate that the SPL values have indeed been reduced. Figure 15 compares the SPL contours between the best case in the dataset and the optimized case, where the actual maximum SPL value of 13.09978 (from AniForm^TM^) is obtained, which is better than the ANN predicted value of 14.409. This corresponds to a 17.4% reduction in maximum SPL.

Some insights were obtained from the ANN outputs, with regard to the double-dome geometry and PW/SAN laminate system. Firstly, the optimized laminate orientation appears to be neither at 0 nor 45 degrees but rather at an angle of about 35 degrees, which is slightly less than 45 degrees. Secondly, lower preload, spring stiffness and ramp rates seemed to be the preferrable conditions to reduce surface defects on the part. Lastly, a finite length of gripping edge seemed to be preferred over point grip.

### 5.3. Optimizing Shear Angle

#### Prediction of Proportion of Nodes with Designated Shear Angle Ranges

It was arbitrarily predetermined that the desired ply shear angle (SA) for the composite laminate falls into the range between 40 to 50 degrees. The understanding was to allow the ply to deform more by shear than in bending, so as to reduce the possibility of part wrinkling, provided that the shear values are below that for shear locking of the warp and weft. The setting of this shear angle coverage depends on the polymer–fabric architecture system of the ply and is typically determined from the stable slope region of force versus shear angle plots of the picture-frame or bias-extension test.

**Figure 16:**
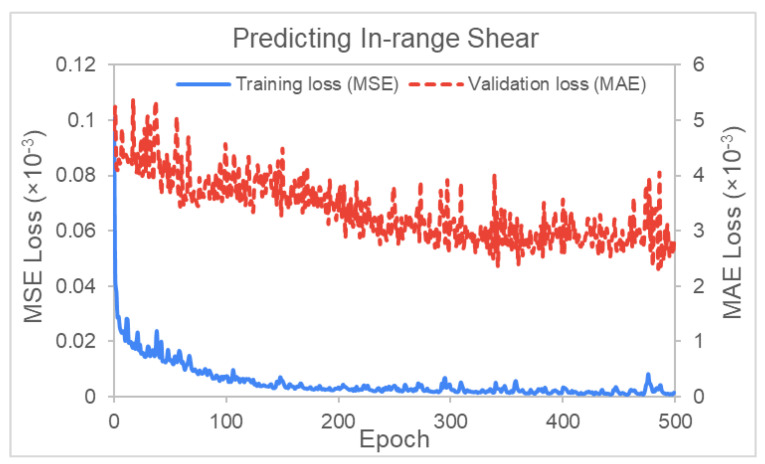
Graph of training and validation loss versus epoch (In-range shear prediction).

In the ANN, any nodes that satisfy this condition are labeled “in-range” while those whose shear value is above 50 degrees are labeled “excessive”. Absolute values are taken since the shear can be positive or negative depending on the shear direction. An ANN was developed to predict the proportion of in-range nodes in the mesh (# of in-range nodes/total # of nodes). As the label values are between 0 and 1 by nature, no normalization or rescaling was required. Figure 16 shows the training loss for the prediction of in-range nodes with shear angles between 40 to 50 degrees. The training and validation simulations were also set to 1000 epochs (training cycles). The figure shows that the training losses significantly reduce from 8 × 10^−5^ to below 1 × 10^−5^ over the first 200 epochs (or training cycles), while validation losses reduced from 5 × 10^−3^ to 2.5 × 10^−3^ over 500 epochs. The losses subsequently stabilize around these values from 500 epochs onwards.

**Figure 17:**
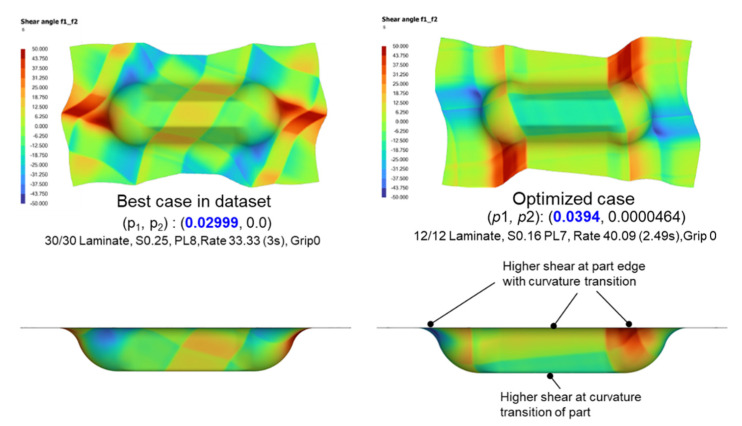
Comparison of shear angle distribution between the best case in the dataset (**left**) and the optimized case (**right**); bottom views (**top**) and side views (**bottom**).

For predicting the proportion of in-range nodes, the best MAE loss was found to be 0.002321. After various runs, the best outputs with their corresponding process parameters are:

$$\left(p_{1},p_{2}\right)=\left(0.0378,0.0063\right);\;lam\;-15/-15,S0.02\;PL8,\;rate\;76.54\;(1.31\;s),\;grip\;0\;(Point).$$

where $p_{1}$ and $p_{2}$ refer to the proportion of in-range and excessive nodes, respectively, and the corresponding process parameters obtained for this case are then used to rerun the AniForm^TM^ thermoforming simulation to obtain the actual shear angle contour to validate the increase in shear angle coverage. In the test dataset, the highest in-range node proportion is 0.02999, with the process parameters as follows: lam 30/30, S0.25 PL8, rate 33.33 (3 s), grip 0 (Point). Its shear angle distribution is shown in Figure 17. The highest *p_1_* value found from the rerun simulation is 0.0394, which is about 30% more than the best case in the dataset. From the shear angle contour plots, it was observed that the process parameters used in the optimized case would result in higher shear around the transition region between the excess material and the final part, which will be beneficial to mitigate wrinkling. The results consistently show that point grips would initiate greater regions of ply shear than edge grips of finite lengths. Hence, the grip type used might affect the maximum split-path length and shear angle coverage in opposite ways.

## 6. Conclusions and Future Directions

Our work has shown that machine learning methods can help in defect analysis and inverse problem tracing, as well as in manufacturing process optimization and in deriving insights. The first ANN is able to analyze a given contour image of split-path length and predicts the laminate orientation, tensioner stiffness and preload parameters reasonably well, with better predictions from SingleVar than MultiVar since the latter requires much larger datasets than what we have provided currently. The second ANN is able to output the optimized process parameters for a reduced split-path length of 17.4% and an increased forming shear angle coverage of 31%. The image analysis method is novel for composite manufacturing and photos from actual parts may be used instead of simulation results, such as through computer vision methods.

This pioneering work creates a foundation to consider other challenging aspects of data-driven models, such as application robustness and training efficiency. The presented approach for optimizing individual metrics allows engineers to find local minima depicting a desirable set of process parameters. A combined target function that takes into account multiple metrics is needed to obtain the global minima for the best set of process parameters. The ANNs could also take into account the manufacturing constraints to provide more useful neural network predictions. In our future work, the developed models will be used in transfer learning to predict and optimize the different thicknesses of laminate and final part geometries rather than the presented double-dome geometry.

## Figures and Tables

**Table 1:** Ply properties used for the plain-woven laminate thermoforming analysis.

General Properties				
Isotropic Density	υ = 0	E = 1 × 10^−16^ MPa	Rho = 2 × 10^−9^	
In-plane Model				
Fiber	10,000 MPa			
Isotropic Elastic	υ = 0	E = 0.02295 MPa		
Mooney Rivlin	C10 = 0	C01 = 0.0072		
Cross-Viscosity	Eta0 = 0.5	EtaInf = 0.04	M = 75	N = −0.17
Bending Model				
Isotropic Elastic	E = 200 MPa			
Cross-Viscosity	Eta0 = 2000 MPa	EtaInf = 10 MPa	M = 7200	N = 0.02

**Table 2:** Combinations of process parameters used.

Laminate Orientation (Deg)	Tensioner Stiffness (N/mm)	Preload (N)	Press Rate (mm/s)	Grip Size (mm)
0, +/− 15, +/−30, +/−45	0.5, 1.0, 1.5, 1.75, 2.0	2, 4, 8	66.7, 33.3, 16.7	0 (point), 2, 4, 8

**Table 3:** The AlexNet architecture adopted for this study.

Layer	No. of Filters/ Neurons	Filter Size	Stride	Padding	Size of Feature Map	Activation Function
Input	-	-	-	-	3 × 224 × 224	-
Conv 1	64	11 × 11	4	-	64 × 54 × 54	ReLU
Max Pool 1	-	3 × 3	2	-	64 × 26 × 26	-
Conv 2	192	5 × 5	1	2	192 × 26 × 26	ReLU
Max Pool 2	-	3 × 3	2	-	192 × 12 × 12	-
Conv 3	384	3 × 3	1	1	384 × 12 × 12	ReLU
Conv 4	256	3 × 3	1	1	256 × 12 ×12	ReLU
Conv 5	256	3 × 3	1	1	256 × 12 × 12	ReLU
Max Pool 3	-	3 × 3	2	-	256 × 6 × 6	-
FC 1	256 × 6 × 6 × 4096	-	-	-	4096	ReLU
FC 2	4096	-	-	-	4096	ReLU
FC 3	4096	-	-	-	1	-

**Table 4:** Summary of training statistics and accuracy for inverse modeling.

Parameters	MultiVar Abs Error	SingleVar Abs Error
Laminate Orientation Angle	9.5°	0.7°
Spring Stiffness of Grip Tensioners	0.32736 N/mm	0.30024 N/mm
Preload of Grip Tensioners	1.77136 N	2.02176 N
Press Interval (=1/Press Rate)	1.9727 s	1.1719 s
Grip Size	1.2785 mm	1.3362 mm
